# Supplementary material for: Studies on the Accumulation of Secondary Metabolites and Evaluation of Biological Activity of In Vitro Cultures of Ruta montana L. in Temporary Immersion Bioreactors
Source: Int J Mol Sci. 2023 Apr 11;24(8):7045. doi: 10.3390/ijms24087045 (PMC10138805; doi:10.3390/ijms24087045)
Supplement: Supplementary file 1 [file ijms-24-07045-s001.zip › ijms-2186575-supplementary.pdf]

Studies on the accumulation of secondary metabolites and evaluation of biological activity of *in vitro* cultures of *Ruta montana* in temporary immersion bioreactors

Supplementary files

I. HPLC ANALYSIS – sample chromatograms

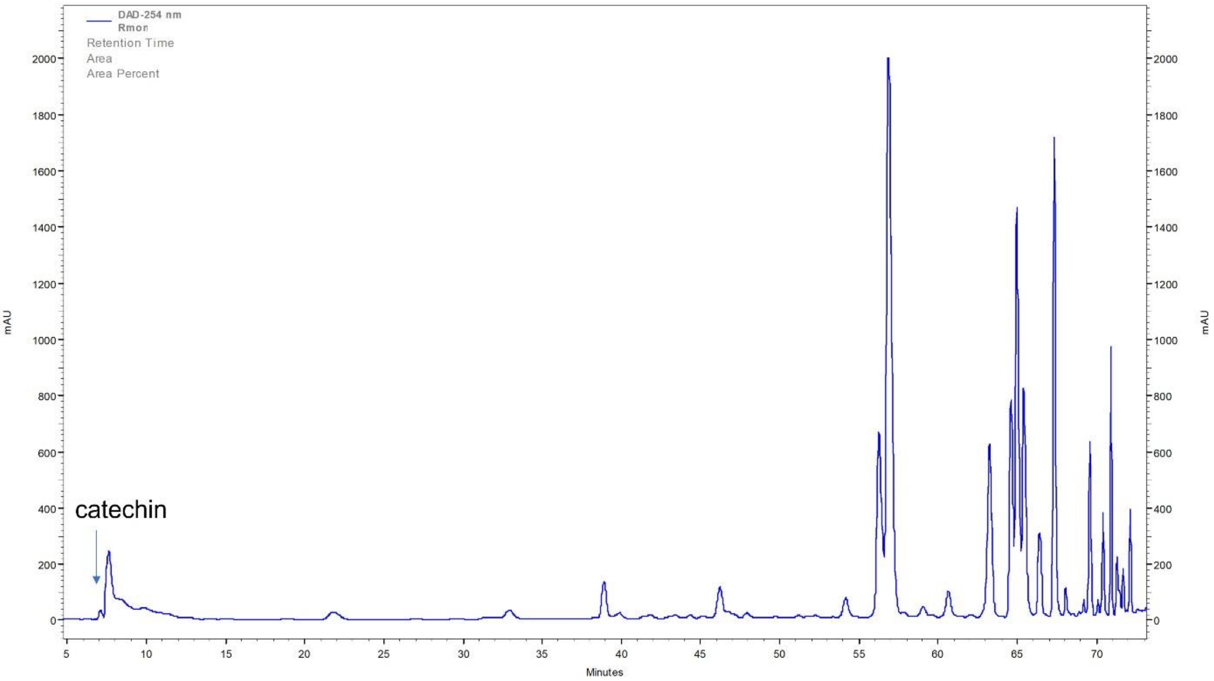

Figure S1 Sample chromatogram of the extract from *Ruta montana in vitro* cultures (0.1/0.1 LS medium, 5-week growth cycle).

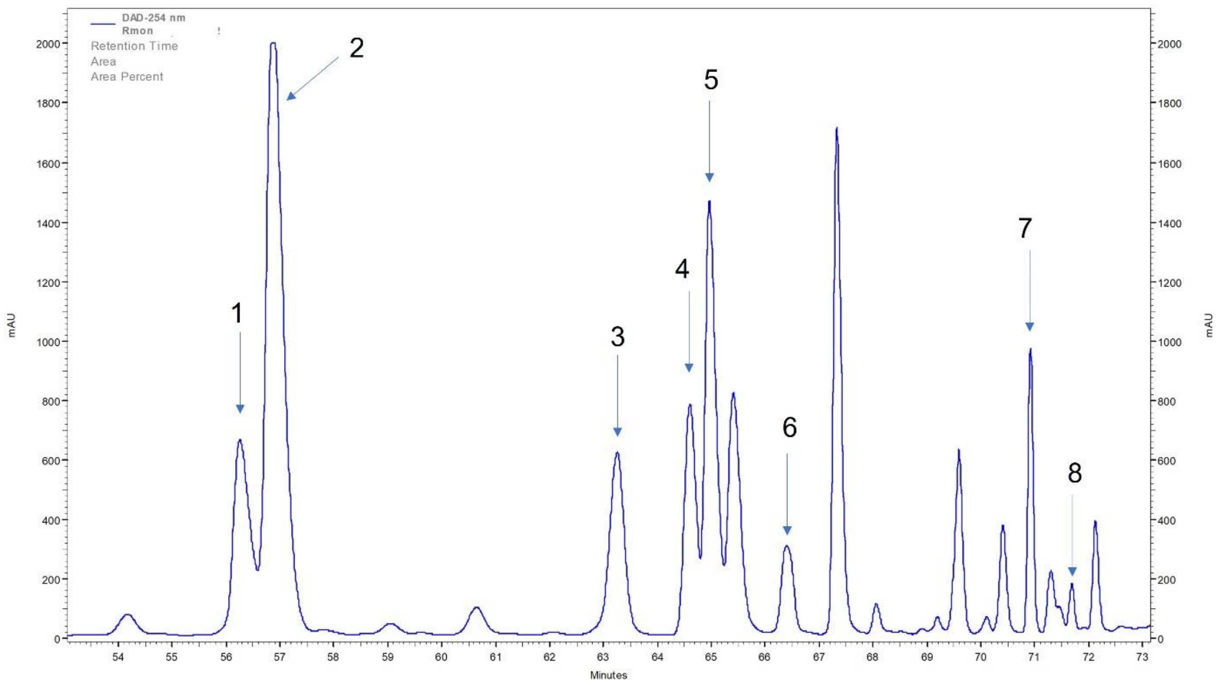

Figure S2 Enlarged fragment of a sample chromatogram of the extract from *Ruta montana in vitro* cultures (0.1/0.1 LS medium, 5-week growth cycle) 1. psoralen, 2. xanthotoxin, 3. isopimpinellin, 4. skimmianine, 5. bergapten, 6. γ-fagarine, 7. 7-isopentenyloxy-γ-fagarine, 8. Isoimperatorin

II. STATISTICAL ANALYSIS

Statistical analysis of the content of individual secondary metabolites and comparison of homogeneous groups. Two-way analysis of variance (ANOVA), NIR post hoc test, dependent variable - metabolite content [mg/100 g DW], independent variables - growth cycle (4, 5 weeks) and LS medium variant (NAA/BAP 0.5/0.5, 0.1/0.5, 0.5/1.0, 0.1/0.1, 1.0/1.0 mg/L).

Results are shown:

1. ANOVA assessment for the metabolite content

2. Significant differences detected by NIR test

3. Homogeneous groups (a,b,c,d,e,f,g) of the results at significance level $\alpha = 0.05$

Abbreviations: SS – sum of squares; d. o f. - degrees of freedom; MS -mean square; F – Fisher's statistic; p – critical level of significance

Xanthotoxin

1.

Source	SS	d.o f.	MS	F	p
week	294	1	294	0.138	0.714027
medium variant	93660	4	23415	11.004	0.000070
week*medium variant	57699	4	14425	6.779	0.001283
Error	42558	20	2128		

2.

No.	NIR test; xanthotoxin content mg/100g											
	week	medium variant	{1} 863.78	{2} 682.89	{3} 885.92	{4} 731.96	{5} 748.75	{6} 772.92	{7} 825.65	{8} 846.06	{9} 771.51	{10} 665.85
1	5	0.1/0.1		0.000108	0.563284	0.002255	0.006261	0.025566	0.323453	0.643061	0.023628	0.000038
2	5	0.1/0.5	0.000108		0.000028	0.207451	0.095709	0.026795	0.001148	0.000324	0.028972	0.655885
3	5	0.5/0.5	0.563284	0.000028		0.000573	0.001622	0.007072	0.125252	0.302543	0.006502	0.000010
4	5	0.5/1	0.002255	0.207451	0.000573		0.660580	0.289778	0.021804	0.006622	0.306171	0.094544
5	5	1.0/1.0	0.006261	0.095709	0.001622	0.660580		0.528353	0.054579	0.017742	0.552355	0.039659
6	6	0.1/0.1	0.025566	0.026795	0.007072	0.289778	0.528353		0.176793	0.066358	0.970635	0.010062
7	6	0.1/0.5	0.323453	0.001148	0.125252	0.021804	0.054579	0.176793		0.593921	0.166061	0.000399
8	6	0.5/0.5	0.643061	0.000324	0.302543	0.006622	0.017742	0.066358	0.593921		0.061720	0.000113
9	6	0.5/1	0.023628	0.028972	0.006502	0.306171	0.552355	0.970635	0.166061	0.061720		0.010928
10	6	1.0/1.0	0.000038	0.655885	0.000010	0.094544	0.039659	0.010062	0.000399	0.000113	0.010928	

3.

No.	NIR test – comparison of homogenous groups			homogenous groups					
	week	medium variant	xanthotoxin content mg/100g DW mean	a	b	c	d	e	f
10	6 weeks	1.0/1.0	665.8516	****					
2	5 weeks	0.1/0.5	682.8892	****	****				
4	5 weeks	0.5/1	731.9577	****	****	****			
5	5 weeks	1.0/1.0	748.7459		****	****	****		
9	6 weeks	0.5/1	771.5113			****	****	****	
6	6 weeks	0.1/0.1	772.9153			****	****	****	
7	6 weeks	0.1/0.5	825.6509				****	****	****
8	6 weeks	0.5/0.5	846.0587					****	****
1	5 weeks	0.1/0.1	863.7813						****
3	5 weeks	0.5/0.5	885.9183						****

Bergapten

1.

Source	SS	d.o f.	MS	F	p
week	6989	1	6989	6.926	0.015988
medium variant	13083	4	3271	3.241	0.033325
week*medium variant	2800	4	700	0.694	0.605085
Error	20181	20	1009		

2.

No.	NIR test; bergapten content mg/100g											
	week	medium variant	{1} 435.32	{2} 375.70	{3} 440.91	{4} 386.32	{5} 445.99	{6} 403.75	{7} 364.99	{8} 395.18	{9} 378.85	{10} 388.82
1	5 weeks	0.1/0.1		0.032415	0.831633	0.073432	0.685321	0.237723	0.013430	0.137367	0.041612	0.088145
2	5 weeks	0.1/0.5	0.032415		0.020590	0.686422	0.013475	0.292186	0.684236	0.461242	0.904302	0.618255
3	5 weeks	0.5/0.5	0.831633	0.020590		0.048157	0.846770	0.167437	0.008334	0.093152	0.026659	0.058319
4	5 weeks	0.5/1	0.073432	0.686422	0.048157		0.032313	0.509136	0.420592	0.736198	0.776400	0.924044
5	5 weeks	1.0/1.0	0.685321	0.013475	0.846770	0.032313		0.119113	0.005360	0.064216	0.017562	0.039409
6	6 weeks	0.1/0.1	0.237723	0.292186	0.167437	0.509136	0.119113		0.150647	0.744387	0.348469	0.571260
7	6 weeks	0.1/0.5	0.013430	0.684236	0.008334	0.420592	0.005360	0.150647		0.258146	0.598931	0.369114
8	6 weeks	0.5/0.5	0.137367	0.461242	0.093152	0.736198	0.064216	0.744387	0.258146		0.536145	0.808898
9	6 weeks	0.5/1	0.041612	0.904302	0.026659	0.776400	0.017562	0.348469	0.598931	0.536145		0.704716
10	6 weeks	1.0/1.0	0.088145	0.618255	0.058319	0.924044	0.039409	0.571260	0.369114	0.808898	0.704716	

3.

No.	NIR test – comparison of homogenous groups			homogenous groups			
	week	medium variant	bergapten content mg/100g DW mean	a	b	c	d
7	6 weeks	0.1/0.5	364.9917	****			
2	5 weeks	0.1/0.5	375.6950	****			
9	6 weeks	0.5/1	378.8531	****			
4	5 weeks	0.5/1	386.3198	****	****		
10	6 weeks	1.0/1.0	388.8240	****	****	****	
8	6 weeks	0.5/0.5	395.1803	****	****	****	****
6	6 weeks	0.1/0.1	403.7548	****	****	****	****
1	5 weeks	0.1/0.1	435.3230		****	****	****
3	5 weeks	0.5/0.5	440.9098			****	****
5	5 weeks	1.0/1.0	445.9873				****

Isopimpinellin

1.

Source	SS	d.o f.	MS	F	p
week	26136.2	1	26136.2	93.920	0.000000
medium variant	23511.5	4	5877.9	21.122	0.000001
week*medium variant	10036.7	4	2509.2	9.017	0.000248
Error	5565.6	20	278.3		

2.

No..	NIR test; isopimpinellin content mg/100g											
	week	medium variant	{1} 81.085	{2} 100.75	{3} 92.888	{4} 90.254	{5} 113.97	{6} 79.504	{7} 148.58	{8} 154.44	{9} 168.38	{10} 223.21
1	5 weeks	0.1/0.1		0.164226	0.396453	0.508574	0.025472	0.908752	0.000076	0.000029	0.000003	0.000000
2	5 weeks	0.1/0.5	0.164226		0.570103	0.449797	0.343504	0.134430	0.002197	0.000806	0.000075	0.000000
3	5 weeks	0.5/0.5	0.396453	0.570103		0.848556	0.137378	0.337519	0.000572	0.000209	0.000020	0.000000
4	5 weeks	0.5/1	0.508574	0.449797	0.848556		0.097023	0.439254	0.000364	0.000133	0.000013	0.000000
5	5 weeks	1.0/1.0	0.025472	0.343504	0.137378	0.097023		0.019900	0.019445	0.007546	0.000713	0.000000
6	6 weeks	0.1/0.1	0.908752	0.134430	0.337519	0.439254	0.019900		0.000058	0.000022	0.000002	0.000000
7	6 weeks	0.1/0.5	0.000076	0.002197	0.000572	0.000364	0.019445	0.000058		0.671567	0.161604	0.000023
8	6 weeks	0.5/0.5	0.000029	0.000806	0.000209	0.000133	0.007546	0.000022	0.671567		0.318450	0.000061
9	6 weeks	0.5/1	0.000003	0.000075	0.000020	0.000013	0.000713	0.000002	0.161604	0.318450		0.000663
10	6 weeks	1.0/1.0	0.000000	0.000000	0.000000	0.000000	0.000000	0.000000	0.000023	0.000061	0.000663	

3.

Nr podkl.	NIR test – comparison of homogenous groups			homogenous groups			
	week	medium variant	isopimpinellin content mg/100g DW mean	a	b	c	d
6	6 weeks	0.1/0.1	79.5042	****			
1	5 weeks	0.1/0.1	81.0852	****			
4	5 weeks	0.5/1	90.2535	****	****		
3	5 weeks	0.5/0.5	92.8884	****	****		
2	5 weeks	0.1/0.5	100.7532	****	****		
5	5 weeks	1.0/1.0	113.9687		****		
7	6 weeks	0.1/0.5	148.5801			****	
8	6 weeks	0.5/0.5	154.4412			****	
9	6 weeks	0.5/1	168.3773			****	
10	6 weeks	1.0/1.0	223.2084				****

Psoralen

1.

Source	SS	d.o f.	MS	F	p
week	1500	1	1500	4.717	0.042071
medium variant	72370	4	18093	56.876	0.000000
week*medium variant	16968	4	4242	13.335	0.000019
Error	6362	20	318		

2.

No..	NIR test; psoralen content mg/100g											
	week	medium variant	{1} 81.085	{2} 100.75	{3} 92.888	{4} 90.254	{5} 113.97	{6} 79.504	{7} 148.58	{8} 154.44	{9} 168.38	{10} 223.21
1	5 weeks	0.1/0.1		0.000000	0.000000	0.000000	0.000000	0.000001	0.000000	0.000000	0.000000	0.000000
2	5 weeks	0.1/0.5	0.000000		0.040376	0.056634	0.358844	0.001293	0.834003	0.980260	0.378035	0.040503
3	5 weeks	0.5/0.5	0.000000	0.040376		0.867536	0.224700	0.000008	0.026008	0.042483	0.211587	0.998781
4	5 weeks	0.5/1	0.000000	0.056634	0.867536		0.291288	0.000012	0.036953	0.059491	0.275326	0.868736
5	5 weeks	1.0/1.0	0.000000	0.358844	0.224700	0.291288		0.000144	0.263106	0.371541	0.970341	0.225252
6	6 weeks	0.1/0.1	0.000001	0.001293	0.000008	0.000012	0.000144		0.002118	0.001220	0.000158	0.000008
7	6 weeks	0.1/0.5	0.000000	0.834003	0.026008	0.036953	0.263106	0.002118		0.814778	0.278549	0.026092
8	6 weeks	0.5/0.5	0.000000	0.980260	0.042483	0.059491	0.371541	0.001220	0.814778		0.391178	0.042616
9	6 weeks	0.5/1	0.000000	0.378035	0.211587	0.275326	0.970341	0.000158	0.278549	0.391178		0.212114
10	6 weeks	1.0/1.0	0.000000	0.040503	0.998781	0.868736	0.225252	0.000008	0.026092	0.042616	0.212114	

3.

No.	NIR test – comparison of homogenous groups			homogenous groups				
	week	medium variant	psoralen content mg/100g DW mean	a	b	c	d	e
3	5 weeks	0.5/0.5	150.2087	****				
10	6 weeks	1.0/1.0	150.2313	****				
4	5 weeks	0.5/1	152.6690	****	****			
5	5 weeks	1.0/1.0	168.4540	****	****	****		
9	6 weeks	0.5/1	169.0022	****	****	****		
8	6 weeks	0.5/0.5	181.7660		****	****		
2	5 weeks	0.1/0.5	182.1309		****	****		
7	6 weeks	0.1/0.5	185.2229			****		
6	6 weeks	0.1/0.1	236.5858				****	
1	5 weeks	0.1/0.1	340.0663					****

Isoimperatorin

1.

Source	SS	d.o f.	MS	F	p
week	4134.8	1	4134.8	42.627	0.000002
medium variant	5611.5	4	1402.9	14.462	0.000011
week*medium variant	2054.9	4	513.7	5.296	0.004482
Error	1940.0	20	97.0		

2.

No.	NIR test; isoimperatorin content mg/100g											
	week	medium variant	{1} 104.01	{2} 79.112	{3} 87.649	{4} 84.004	{5} 105.78	{6} 86.439	{7} 56.352	{8} 35.225	{9} 82.811	{10} 82.326
1	5 weeks	0.1/0.1		0.005701	0.055413	0.021810	0.827575	0.040989	0.000009	0.000000	0.015848	0.013898
2	5 weeks	0.1/0.5	0.005701		0.301112	0.549884	0.003445	0.373136	0.010338	0.000024	0.650571	0.693651
3	5 weeks	0.5/0.5	0.055413	0.301112		0.655228	0.035508	0.881889	0.000906	0.000002	0.554176	0.515618
4	5 weeks	0.5/1	0.021810	0.549884	0.655228		0.013535	0.765174	0.002599	0.000006	0.883547	0.836888
5	5 weeks	1.0/1.0	0.827575	0.003445	0.035508	0.013535		0.025956	0.000005	0.000000	0.009755	0.008530
6	6 weeks	0.1/0.1	0.040989	0.373136	0.881889	0.765174	0.025956		0.001287	0.000003	0.656735	0.614691
7	6 weeks	0.1/0.5	0.000009	0.010338	0.000906	0.002599	0.000005	0.001287		0.016147	0.003658	0.004198
8	6 weeks	0.5/0.5	0.000000	0.000024	0.000002	0.000006	0.000000	0.000003	0.016147		0.000009	0.000010
9	6 weeks	0.5/1	0.015848	0.650571	0.554176	0.883547	0.009755	0.656735	0.003658	0.000009		0.952578
10	6 weeks	1.0/1.0	0.013898	0.693651	0.515618	0.836888	0.008530	0.614691	0.004198	0.000010	0.952578	

3.

No.	NIR test – comparison of homogenous groups			homogenous groups				
	week	medium variant	7-isoimperatorin content mg/100g DW mean	a	b	c	d	e
8	6 weeks	0.5/0.5	35.2251	****				
7	6 weeks	0.1/0.5	56.3519		****			
2	5 weeks	0.1/0.5	79.1125			****		
10	6 weeks	1.0/1.0	82.3263			****		
9	6 weeks	0.5/1	82.8105			****		
4	5 weeks	0.5/1	84.0036			****		
6	6 weeks	0.1/0.1	86.4385			****		
3	5 weeks	0.5/0.5	87.6487			****	****	
1	5 weeks	0.1/0.1	104.0066				****	****
5	5 weeks	1.0/1.0	105.7812					****

Total furanocoumarins

1.

Source	SS	d.o f.	MS	F	p
week	1774	1	1774	0.42	0.525305
medium variant	182674	4	45668	10.76	0.000081
week*medium variant	161292	4	40323	9.50	0.000179
Error	84879	20	4244		

2.

No..	NIR test; furanocoumarin total content content mg/100g											
	week	medium variant	{1} 1824.3	{2} 1420.6	{3} 1657.6	{4} 1445.2	{5} 1582.9	{6} 1579.2	{7} 1580.8	{8} 1612.7	{9} 1570.6	{10} 1510.4
1	5 weeks	0.1/0.1		0.000000	0.005228	0.000001	0.000201	0.000170	0.000183	0.000741	0.000117	0.000009
2	5 weeks	0.1/0.5	0.000000		0.000243	0.648424	0.006288	0.007368	0.006886	0.001742	0.010589	0.106674
3	5 weeks	0.5/0.5	0.005228	0.000243		0.000716	0.175898	0.156191	0.164388	0.408557	0.117485	0.011916
4	5 weeks	0.5/1	0.000001	0.648424	0.000716		0.017524	0.020383	0.019110	0.005057	0.028751	0.234262
5	5 weeks	1.0/1.0	0.000201	0.006288	0.175898	0.017524		0.944665	0.968312	0.582363	0.818287	0.188055
6	6 weeks	0.1/0.1	0.000170	0.007368	0.156191	0.020383	0.944665		0.976318	0.536281	0.872535	0.210870
7	6 weeks	0.1/0.5	0.000183	0.006886	0.164388	0.019110	0.968312	0.976318		0.555746	0.849238	0.200861
8	6 weeks	0.5/0.5	0.000741	0.001742	0.408557	0.005057	0.582363	0.536281	0.555746		0.437768	0.068978
9	6 weeks	0.5/1	0.000117	0.010589	0.117485	0.028751	0.818287	0.872535	0.849238	0.437768		0.271793
10	6 weeks	1.0/1.0	0.000009	0.106674	0.011916	0.234262	0.188055	0.210870	0.200861	0.068978	0.271793	

3.

No.	NIR test – comparison of homogenous groups			homogenous groups			
	week	medium variant	coumarin total content mg/100g DW mean	a	b	c	d
2	5 weeks	0.1/0.5	1420.581	****			
4	5 weeks	0.5/1	1445.204	****			
10	6 weeks	1.0/1.0	1510.442	****	****		
9	6 weeks	0.5/1	1570.555		****	****	
6	6 weeks	0.1/0.1	1579.199		****	****	
7	6 weeks	0.1/0.5	1580.797		****	****	
5	5 weeks	1.0/1.0	1582.937		****	****	
8	6 weeks	0.5/0.5	1612.671		****	****	
3	5 weeks	0.5/0.5	1657.574			****	
1	5 weeks	0.1/0.1	1824.262				****

Skimmianine

1.

Source	SS	d.o f.	MS	F	p
week	49454.1	1	49454.1	131.854	0.000000
medium variant	29916.3	4	7479.1	19.941	0.000001
week*medium variant	16018.1	4	4004.5	10.677	0.000085
Error	7501.3	20	375.1		

2.

No..	NIR test; skimmianine content mg/100g											
	week	medium variant	{1} 225.46	{2} 197.64	{3} 195.22	{4} 233.73	{5} 179.84	{6} 126.58	{7} 89.379	{8} 51.950	{9} 199.69	{10} 158.27
1	5 weeks	0.1/0.1		0.093859	0.070317	0.606445	0.009156	0.000004	0.000000	0.000000	0.118920	0.000393
2	5 weeks	0.1/0.5	0.093859		0.880018	0.033543	0.273524	0.000222	0.000001	0.000000	0.897981	0.021712
3	5 weeks	0.5/0.5	0.070317	0.880018		0.024362	0.342154	0.000317	0.000002	0.000000	0.780282	0.029973
4	5 weeks	0.5/1	0.606445	0.033543	0.024362		0.002787	0.000001	0.000000	0.000000	0.043738	0.000116
5	5 weeks	1.0/1.0	0.009156	0.273524	0.342154	0.002787		0.003059	0.000013	0.000000	0.223668	0.187835
6	6 weeks	0.1/0.1	0.000004	0.000222	0.000317	0.000001	0.003059		0.028991	0.000131	0.000164	0.058770
7	6 weeks	0.1/0.5	0.000000	0.000001	0.000002	0.000000	0.000013	0.028991		0.028131	0.000001	0.000305
8	6 weeks	0.5/0.5	0.000000	0.000000	0.000000	0.000000	0.000000	0.000131	0.028131		0.000000	0.000002
9	6 weeks	0.5/1	0.118920	0.897981	0.780282	0.043738	0.223668	0.000164	0.000001	0.000000		0.016422
10	6 weeks	1.0/1.0	0.000393	0.021712	0.029973	0.000116	0.187835	0.058770	0.000305	0.000002	0.016422	

3.

No.	NIR test – comparison of homogenous groups			homogenous groups						
	week	medium variant	skimmianine content mg/100g DW mean	a	b	c	d	e	f	g
8	6 weeks	0.5/0.5	51.9495	****						
7	6 weeks	0.1/0.5	89.3789		****					
6	6 weeks	0.1/0.1	126.5806			****				
10	6 weeks	1.0/1.0	158.2740			****	****			
5	5 weeks	1.0/1.0	179.8368				****	****		
3	5 weeks	0.5/0.5	195.2234					****	****	
2	5 weeks	0.1/0.5	197.6410					****	****	
9	6 weeks	0.5/1	199.6943					****	****	
1	5 weeks	0.1/0.1	225.4566						****	****
4	5 weeks	0.5/1	233.7330							****

γ-fagarine

1.

Source	SS	d.o f.	MS	F	p
week	3386.9	1	3386.9	10.906	0.003556
medium variant	69632.0	4	17408.0	56.055	0.000000
week*medium variant	8975.7	4	2243.9	7.226	0.000905
Error	6211.0	20	310.6		

2.

No.	NIR test; γ-fagarine content mg/100g											
	week	medium variant	{1} 305.42	{2} 172.27	{3} 146.64	{4} 133.85	{5} 133.61	{6} 215.82	{7} 159.87	{8} 151.05	{9} 128.08	{10} 130.70
1	5 weeks	0.1/0.1		0.000000	0.000000	0.000000	0.000000	0.000004	0.000000	0.000000	0.000000	0.000000
2	5 weeks	0.1/0.5	0.000000		0.090129	0.014713	0.014184	0.006655	0.399253	0.155962	0.006032	0.009076
3	5 weeks	0.5/0.5	0.000000	0.090129		0.384540	0.375796	0.000107	0.368783	0.762357	0.211807	0.280989
4	5 weeks	0.5/1	0.000000	0.014713	0.384540		0.986765	0.000014	0.085577	0.245834	0.692798	0.828879
5	5 weeks	1.0/1.0	0.000000	0.014184	0.375796	0.986765		0.000014	0.082916	0.239486	0.705009	0.841810
6	6 weeks	0.1/0.1	0.000004	0.006655	0.000107	0.000014	0.000014		0.000913	0.000218	0.000006	0.000009
7	6 weeks	0.1/0.5	0.000000	0.399253	0.368783	0.085577	0.082916	0.000913		0.546803	0.038974	0.056139
8	6 weeks	0.5/0.5	0.000000	0.155962	0.762357	0.245834	0.239486	0.000218	0.546803		0.126073	0.172568
9	6 weeks	0.5/1	0.000000	0.006032	0.211807	0.692798	0.705009	0.000006	0.038974	0.126073		0.857540
10	6 weeks	1.0/1.0	0.000000	0.009076	0.280989	0.828879	0.841810	0.000009	0.056139	0.172568	0.857540	

3.

No.	NIR test – comparison of homogenous groups			homogenous groups				
	week	medium variant	γ-fagarine content mg/100g DW mean	a	b	c	d	e
9	6 weeks	0.5/1	128.0828	****				
10	6 weeks	1.0/1.0	130.6992	****	****			
5	5 weeks	1.0/1.0	133.6085	****	****			
4	5 weeks	0.5/1	133.8502	****	****			
3	5 weeks	0.5/0.5	146.6426	****	****	****		
8	6 weeks	0.5/0.5	151.0534	****	****	****		
7	6 weeks	0.1/0.5	159.8733		****	****		
2	5 weeks	0.1/0.5	172.2670			****		
6	6 weeks	0.1/0.1	215.8248				****	
1	5 weeks	0.1/0.1	305.4182					****

7-isopentenyloxy-γ-fagarine

1.

Source	SS	d.o f.	MS	F	p
week	583.84	1	583.84	79.827	0.000000
medium variant	657.49	4	164.37	22.474	0.000000
week*medium variant	330.76	4	82.69	11.306	0.000058
Error	146.28	20	7.31		

2.

No.	NIR test; 7-isopentenyloxy-γ-fagarine content mg/100g											
	week	medium variant	{1} 30.787	{2} 18.594	{3} 26.169	{4} 21.925	{5} 25.901	{6} 39.417	{7} 30.658	{8} 42.199	{9} 32.882	{10} 22.335
1	5 weeks	0.1/0.1		0.000021	0.049508	0.000682	0.038723	0.000872	0.954289	0.000047	0.354009	0.001053
2	5 weeks	0.1/0.5	0.000021		0.002647	0.147046	0.003503	0.000000	0.000024	0.000000	0.000003	0.105729
3	5 weeks	0.5/0.5	0.049508	0.002647		0.068946	0.904385	0.000007	0.055555	0.000001	0.006469	0.097864
4	5 weeks	0.5/1	0.000682	0.147046	0.068946		0.086882	0.000000	0.000782	0.000000	0.000075	0.854538
5	5 weeks	1.0/1.0	0.038723	0.003503	0.904385	0.086882		0.000006	0.043569	0.000000	0.004909	0.122022
6	6 weeks	0.1/0.1	0.000872	0.000000	0.000007	0.000000	0.000006		0.000761	0.222151	0.007752	0.000000
7	6 weeks	0.1/0.5	0.954289	0.000024	0.055555	0.000782	0.043569	0.000761		0.000041	0.326000	0.001206
8	6 weeks	0.5/0.5	0.000047	0.000000	0.000001	0.000000	0.000000	0.222151	0.000041		0.000421	0.000000
9	6 weeks	0.5/1	0.354009	0.000003	0.006469	0.000075	0.004909	0.007752	0.326000	0.000421		0.000115
10	6 weeks	1.0/1.0	0.001053	0.105729	0.097864	0.854538	0.122022	0.000000	0.001206	0.000000	0.000115	

3.

No.	NIR test– comparison of homogenous groups			homogenous groups				
	week	medium variant	7-isopentenyloxy-γ-fagarine content mg/100g DW mean	a	b	c	d	e
2	5 weeks	0.1/0.5	18.59395	****				
4	5 weeks	0.5/1	21.92506	****	****			
10	6 weeks	1.0/1.0	22.33515	****	****			
5	5 weeks	1.0/1.0	25.90084		****			
3	5 weeks	0.5/0.5	26.16948		****	****		
7	6 weeks	0.1/0.5	30.65834			****	****	
1	5 weeks	0.1/0.1	30.78651				****	
9	6 weeks	0.5/1	32.88177				****	
6	6 weeks	0.1/0.1	39.41656					****
8	6 weeks	0.5/0.5	42.19896					****

Total furoquinoline alkaloids

1.

Source	SS	d.o f.	MS	F	p
week	65750	1	65750	140.810	0.000000
medium variant	104383	4	26096	55.887	0.000000
week*medium variant	25511	4	6378	13.659	0.000016
Error	9339	20	467		

2.

No.	NIR test; alkaloid total content content mg/100g											
	week	medium variant	{1} 561.66	{2} 388.50	{3} 368.04	{4} 389.51	{5} 339.35	{6} 381.82	{7} 279.91	{8} 245.20	{9} 360.66	{10} 311.31
1	5 weeks	0.1/0.1		0.000000	0.000000	0.000000	0.000000	0.000000	0.000000	0.000000	0.000000	0.000000
2	5 weeks	0.1/0.5	0.000000		0.259709	0.955081	0.011403	0.708970	0.000005	0.000000	0.130231	0.000293
3	5 weeks	0.5/0.5	0.000000	0.259709		0.237756	0.119592	0.443722	0.000070	0.000001	0.680335	0.004343
4	5 weeks	0.5/1	0.000000	0.955081	0.237756		0.010050	0.667761	0.000005	0.000000	0.117664	0.000256
5	5 weeks	1.0/1.0	0.000000	0.011403	0.119592	0.010050		0.025840	0.003054	0.000032	0.241152	0.127714
6	6 weeks	0.1/0.1	0.000000	0.708970	0.443722	0.667761	0.025840		0.000012	0.000000	0.244357	0.000709
7	6 weeks	0.1/0.5	0.000000	0.000005	0.000070	0.000005	0.003054	0.000012		0.063185	0.000183	0.090348
8	6 weeks	0.5/0.5	0.000000	0.000000	0.000001	0.000000	0.000032	0.000000	0.063185		0.000002	0.001271
9	6 weeks	0.5/1	0.000000	0.130231	0.680335	0.117664	0.241152	0.244357	0.000183	0.000002		0.011128
10	6 weeks	1.0/1.0	0.000000	0.000293	0.004343	0.000256	0.127714	0.000709	0.090348	0.001271	0.011128	

3.

No.	NIR test – comparison of homogenous groups			homogenous groups					
	week	medium variant	alkaloid total content mg/100g DW mean	a	b	c	d	e	f
8	6 weeks	0.5/0.5	245.2019	****					
7	6 weeks	0.1/0.5	279.9105	****	****				
10	6 weeks	1.0/1.0	311.3083		****	****			
5	5 weeks	1.0/1.0	339.3462			****	****		
9	6 weeks	0.5/1	360.6589				****	****	
3	5 weeks	0.5/0.5	368.0355				****	****	
6	6 weeks	0.1/0.1	381.8220					****	
2	5 weeks	0.1/0.5	388.5019					****	
4	5 weeks	0.5/1	389.5083					****	
1	5 weeks	0.1/0.1	561.6613						****

Catechin

1.

Source	SS	d.o f.	MS	F	p
week	17540.2	1	17540.2	170.162	0.000000
medium variant	14465.1	4	3616.3	35.082	0.000000
week*medium variant	4443.4	4	1110.9	10.777	0.000080
Error	2061.6	20	103.1		

2.

No.	NIR test; catechin content mg/100g											
	week	medium variant	{1} 179.24	{2} 152.24	{3} 119.40	{4} 122.31	{5} 147.12	{6} 101.60	{7} 118.50	{8} 92.753	{9} 44.014	{10} 121.65
1	5 weeks	0.1/0.1		0.003946	0.000001	0.000001	0.000944	0.000000	0.000000	0.000000	0.000000	0.000001
2	5 weeks	0.1/0.5	0.003946		0.000770	0.001746	0.544174	0.000006	0.000597	0.000001	0.000000	0.001450
3	5 weeks	0.5/0.5	0.000001	0.000770		0.729306	0.003231	0.044197	0.914288	0.004350	0.000000	0.789159
4	5 weeks	0.5/1	0.000001	0.001746	0.729306		0.007184	0.021307	0.650523	0.001938	0.000000	0.937092
5	5 weeks	1.0/1.0	0.000944	0.544174	0.003231	0.007184		0.000022	0.002513	0.000002	0.000000	0.005998
6	6 weeks	0.1/0.1	0.000000	0.000006	0.044197	0.021307	0.000022		0.054947	0.298650	0.000001	0.025247
7	6 weeks	0.1/0.5	0.000000	0.000597	0.914288	0.650523	0.002513	0.054947		0.005575	0.000000	0.707941
8	6 weeks	0.5/0.5	0.000000	0.000001	0.004350	0.001938	0.000002	0.298650	0.005575		0.000009	0.002332
9	6 weeks	0.5/1	0.000000	0.000000	0.000000	0.000000	0.000000	0.000001	0.000000	0.000009		0.000000
10	6 weeks	1.0/1.0	0.000001	0.001450	0.789159	0.937092	0.005998	0.025247	0.707941	0.002332	0.000000	

3.

No.	NIR test – comparison of homogenous groups			homogenous groups					
	week	medium variant	catechin content mg/100g DW mean	a	b	c	d	e	f
9	6 weeks	0.5/1	44.0145	****					
8	6 weeks	0.5/0.5	92.7534		****				
6	6 weeks	0.1/0.1	101.5992		****	****			
7	6 weeks	0.1/0.5	118.4974			****	****		
3	5 weeks	0.5/0.5	119.4009				****		
10	6 weeks	1.0/1.0	121.6476				****		
4	5 weeks	0.5/1	122.3101				****		
5	5 weeks	1.0/1.0	147.1235					****	
2	5 weeks	0.1/0.5	152.2385					****	
1	5 weeks	0.1/0.1	179.2394						****

Statistical analysis of total polyphenols (expressed as mg GAE/g dry extract), total flavonoid (expressed as mg QE/g dry extract) and condensed tannin (expressed as mg CE/g dry extract) content as well as the chelating activity, reported as IC50, and reducing power, reported as ASE/mL, of the different extracts. One-way analysis of variance (ANOVA), Turkey post hoc test, dependent variable - LS medium variant (NAA/BAP 0.5/0.5, 0.1/0.5, 0.5/1.0, 0.1/0.1, 1.0/1.0 mg/L).

Results are shown:

1. ANOVA assessment for LS variant

2. Significant differences detected by Tukey test ($p = 0.05$)

Abbreviations: SS – sum of squares; d. o f. - degrees of freedom; MS -mean square; F – Fisher's statistic; p – critical level of significance

Polyphenol content (mg GAE/g)

1. Anova

Source	SS	d.o f.	MS	F	p
**Treatment (between columns)**	762,4	4	190,6	428,5	P<0,0001
**Residual (within columns)**	11,12	25	0,4448		
**Total**	773,5	29			

2. Tukey's multiple comparisons test

LS variant	Mean Diff,	95,00% CI of diff,		P Value
0.1/0.1 vs 0.1/0.5	-14,67	-15,80 to -13,54	****	<0,0001
0.1/0.1 vs 0.5/0.5	-2,708	-3,838 to -1,577	****	<0,0001
0.1/0.1 vs 0.5/1.0	-4,167	-5,297 to -3,036	****	<0,0001
0.1/0.1 vs 1.0/1.0	-7,386	-8,517 to -6,256	****	<0,0001
0.1/0.5 vs 0.5/0.5	11,96	10,83 to 13,09	****	<0,0001
0.1/0.5 vs. 0.5/1.0	10,50	9,372 to 11,63	****	<0,0001
0.1/0.5 vs. 1.0/1.0	7,283	6,153 to 8,414	****	<0,0001
0.5/0.5 vs. 0.5/1.0	-1,459	-2,590 to -0,3284	**	0,0069
0.5/0.5 vs. 1.0/1.0	-4,679	-5,810 to -3,548	****	<0,0001
0.5/1.0 vs. 1.0/1.0	-3,220	-4,351 to -2,089	****	<0,0001

Flavonoid content (mg QE7g dry extract)

1. Anova

Source	SS	d.o f.	MS	F	p
**Treatment (between columns)**	2732	4	683,0	2004	P<0,0001
**Residual (within columns)**	8,518	25	0,3407		
**Total**	2740	29			

2. Tukey's multiple comparisons test

LS variant	Mean Diff,	95,00% CI of diff,		P Value
0.1/0.1 vs 0.1/0.5	-21,06	-22,05 to -20,07	****	<0,0001
0.1/0.1 vs 0.5/0.5	7,637	6,647 to 8,627	****	<0,0001
0.1/0.1 vs 0.5/1.0	-3,840	-4,830 to -2,851	****	<0,0001
0.1/0.1 vs 1.0/1.0	-8,403	-9,392 to -7,413	****	<0,0001
0.1/0.5 vs 0.5/0.5	28,69	27,70 to 29,68	****	<0,0001
0.1/0.5 vs. 0.5/1.0	17,22	16,23 to 18,20	****	<0,0001
0.1/0.5 vs. 1.0/1.0	12,65	11,66 to 13,64	****	<0,0001
0.5/0.5 vs. 0.5/1.0	-11,48	-12,47 to -10,49	****	<0,0001
0.5/0.5 vs. 1.0/1.0	-16,04	-17,03 to -15,05	****	<0,0001
0.5/1.0 vs. 1.0/1.0	-4,562	-5,552 to -3,572	****	<0,0001

Tannin content

1. Anova

Source	SS	d.o f.	MS	F	p
**Treatment (between columns)**	177,7	4	44,42	241,6	P<0,0001
**Residual (within columns)**	4,596	25	0,1838		
**Total**	182,3	29			

2. Tukey's multiple comparisons test

LS variant	Mean Diff,	95,00% CI of diff,		P Value
0.1/0.1 vs 0.1/0.5	1,586	0,8594 to 2,313	****	<0,0001
0.1/0.1 vs 0.5/0.5	6,260	5,533 to 6,987	****	<0,0001
0.1/0.1 vs 0.5/1.0	5,575	4,848 to 6,302	****	<0,0001
0.1/0.1 vs 1.0/1.0	4,881	4,154 to 5,608	****	<0,0001
0.1/0.5 vs 0.5/0.5	4,673	3,946 to 5,400	****	<0,0001
0.1/0.5 vs. 0.5/1.0	3,989	3,262 to 4,716	****	<0,0001
0.1/0.5 vs. 1.0/1.0	3,295	2,568 to 4,022	****	<0,0001
0.5/0.5 vs. 0.5/1.0	-0,6849	-1,412 to 0,04212	ns	0,0718
0.5/0.5 vs. 1.0/1.0	-1,379	-2,106 to -0,6516	****	<0,0001
0.5/1.0 vs. 1.0/1.0	-0,6938	-1,421 to 0,03326	ns	0,0666

Chelantig activity (IC50)

1.Anova

Source	SS	d.o f.	MS	F	p
**Treatment (between columns)**	20,82	5	4,165		
**Residual (within columns)**	0,05158	30	0,001719	2423	P<0,0001
**Total**	20,88	35			

2.Tukey's multiple comparisons test

LS variant	Mean Diff,	95,00% CI of diff,		P Value
0.1/0.1 vs 0.1/0.5	-1,570	-1,643 to -1,497	****	<0,0001
0.1/0.1 vs 0.5/0.5	-0,04125	-0,1141 to 0,03157	ns	0,5280
0.1/0.1 vs 0.5/1.0	-0,03197	-0,1048 to 0,04084	ns	0,7633
0.1/0.1 vs 1.0/1.0	-0,7875	-0,8603 to -0,7147	****	<0,0001
0.1/0.1 vs BHT	0,8843	0,8115 to 0,9571	****	<0,0001
0.1/0.5 vs 0.5/0.5	1,529	1,456 to 1,601	****	<0,0001
0.1/0.5 vs. 0.5/1.0	1,538	1,465 to 1,611	****	<0,0001
0.1/0.5 vs. 1.0/1.0	0,7823	0,7095 to 0,8551	****	<0,0001
0.1/0.5 vs. BHT	2,454	2,381 to 2,527	****	<0,0001
0.5/0.5 vs. 0.5/1.0	0,009275	-0,06354 to 0,08209	ns	0,9987
0.5/0.5 vs. 1.0/1.0	-0,7463	-0,8191 to -0,6735	****	<0,0001
0.5/0.5 t vs. BHT	0,9256	0,8528 to 0,9984	****	<0,0001
0.5/1.0 vs. 1.0/1.0	-0,7556	-0,8284 to -0,6827	****	<0,0001
0.5/1.0 vs. BHT	0,9163	0,8435 to 0,9891	****	<0,0001
1.0/1.0 vs. BHT	1,672	1,599 to 1,745	****	<0,0001

Reducing power (ASE/mL)

1. Anova

Source	SS	d.o f.	MS	F	p
**Treatment (between columns)**	2618	6	436,3	3,815	0,0026

**Residual (within columns)**	7434	65	114,4	
**Total**	10052	71		

2. Tukey's multiple comparisons test

LS variant	Mean Diff,	95,00% CI of diff,		P Value
0.1/0.1 vs 0.1/0.5	2,333	-0,3389 to 5,005	ns	0,1148
0.1/0.1 vs 0.5/0.5	9,416	6,744 to 12,09	****	<0,0001
0.1/0.1 vs 0.5/1.0	-10,79	-13,46 to -8,120	****	<0,0001
0.1/0.1 vs 1.0/1.0	10,67	7,999 to 13,34	****	<0,0001
0.1/0.1 vs BHT	27,51	24,84 to 30,19	****	<0,0001
0.1/0.5 vs 0.5/0.5	7,083	4,411 to 9,755	****	<0,0001
0.1/0.5 vs. 0.5/1.0	-13,13	-15,80 to -10,45	****	<0,0001
0.1/0.5 vs. 1.0/1.0	8,338	5,666 to 11,01	****	<0,0001
0.1/0.5 vs. BHT	25,18	22,51 to 27,85	****	<0,0001
0.5/0.5 vs. 0.5/1.0	-20,21	-22,88 to -17,54	****	<0,0001
0.5/0.5 vs. 1.0/1.0	1,255	-1,418 to 3,927	ns	0,7102
0.5/0.5 t vs. BHT	18,10	15,43 to 20,77	****	<0,0001
0.5/1.0 vs. 1.0/1.0	21,46	18,79 to 24,14	****	<0,0001
0.5/1.0 vs. BHT	38,31	35,63 to 40,98	****	<0,0001
1.0/1.0 vs. BHT	16,84	14,17 to 19,52	****	<0,0001

Statistical analysis of the content of antioxidant activity expressed by Radical scavenging activity (%) , reducing power (abs 700 nm) and Chelating activity (%) Two-way analysis of variance (ANOVA), Tukey post hoc test, dependent variable - % of activity and abs 700nm - independent variables - LS medium variant (NAA/BAP 0.5/0.5, 0.1/0.5, 0.5/1.0, 0.1/0.1, 1.0/1.0 mg/L).

Results are shown:

1. ANOVA assessment for the metabolite content

2. Significant differences detected by Tukey test (p = 0.05)

Abbreviations: SS – sum of squares; d. o f. - degrees of freedom; MS -mean square; F – Fisher's statistic; p – critical level of significance

Radical scavenging activity (%)

1. Anova

Source	SS	d.o f.	MS	F	p
**Interaction**	520,0	28	18,57	46,18	P<0,0001
**Row Factor**	8755	7	1251	3110	P<0,0001
**Column Factor**	1372	4	342,9	852,7	P<0,0001
**Residual**	32,17	80	0,4022		

2. Tukey's multiple comparisons test

	LS variant	Mean Diff,	95,00% CI of diff,		P Value
0.0625 mg/mL	0.1/0.1 vs 0.1/0.5	1,781	0,3363 to 3,227	**	0,0080
	0.1/0.1 vs 0.5/0.5	1,781	0,3363 to 3,227	**	0,0080
	0.1/0.1 vs 0.5/1.0	1,781	0,3363 to 3,227	**	0,0080
	0.1/0.1 vs 1.0/1.0	1,781	0,3363 to 3,227	**	0,0080
	0.1/0.5 vs 0.5/0.5	0,000	-1,445 to 1,445	ns	>0,9999
	0.1/0.5 vs. 0.5/1.0	0,000	-1,445 to 1,445	ns	>0,9999
	0.1/0.5 vs. 1.0/1.0	0,000	-1,445 to 1,445	ns	>0,9999
	0.5/0.5 vs. 0.5/1.0	0,000	-1,445 to 1,445	ns	>0,9999
	0.5/0.5 vs. 1.0/1.0	0,000	-1,445 to 1,445	ns	>0,9999
	0.5/1.0 vs. 1.0/1.0	0,000	-1,445 to 1,445	ns	>0,9999
0.125 mg/mL	0.1/0.1 vs 0.1/0.5	-1,395	-2,841 to 0,04977	ns	0,0636
	0.1/0.1 vs 0.5/0.5	0,02762	-1,418 to 1,473	ns	>0,9999
	0.1/0.1 vs 0.5/1.0	2,494	1,049 to 3,939	****	<0,0001
	0.1/0.1 vs 1.0/1.0	2,494	1,049 to 3,939	****	<0,0001
	0.1/0.5 vs 0.5/0.5	1,423	-0,02215 to 2,868	ns	0,0557
	0.1/0.5 vs. 0.5/1.0	3,889	2,444 to 5,335	****	<0,0001
	0.1/0.5 vs. 1.0/1.0	3,889	2,444 to 5,335	****	<0,0001
	0.5/0.5 vs. 0.5/1.0	2,466	1,021 to 3,912	****	<0,0001
	0.5/0.5 vs. 1.0/1.0	2,466	1,021 to 3,912	****	<0,0001
	0.5/1.0 vs. 1.0/1.0	0,000	-1,445 to 1,445	ns	>0,9999
0.250 mg/mL	0.1/0.1 vs 0.1/0.5	-0,3559	-1,801 to 1,089	ns	0,9587
	0.1/0.1 vs 0.5/0.5	1,257	-0,1877 to 2,703	ns	0,1183
	0.1/0.1 vs 0.5/1.0	5,819	4,374 to 7,265	****	<0,0001
	0.1/0.1 vs 1.0/1.0	5,819	4,374 to 7,265	****	<0,0001
	0.1/0.5 vs 0.5/0.5	1,613	0,1682 to 3,059	*	0,0209
	0.1/0.5 vs. 0.5/1.0	6,175	4,730 to 7,621	****	<0,0001
	0.1/0.5 vs. 1.0/1.0	6,175	4,730 to 7,621	****	<0,0001
	0.5/0.5 vs. 0.5/1.0	4,562	3,117 to 6,007	****	<0,0001
	0.5/0.5 vs. 1.0/1.0	4,562	3,117 to 6,007	****	<0,0001
	0.5/1.0 vs. 1.0/1.0	0,000	-1,445 to 1,445	ns	>0,9999
0.500 mg/mL	0.1/0.1 vs 0.1/0.5	1,290	-0,1555 to 2,735	ns	0,1030
	0.1/0.1 vs 0.5/0.5	2,159	0,7139 to 3,604	***	0,0007
	0.1/0.1 vs 0.5/1.0	5,632	4,187 to 7,077	****	<0,0001
	0.1/0.1 vs 1.0/1.0	7,389	5,944 to 8,834	****	<0,0001
	0.1/0.5 vs 0.5/0.5	0,8694	-0,5758 to 2,315	ns	0,4526
	0.1/0.5 vs. 0.5/1.0	4,342	2,897 to 5,787	****	<0,0001
	0.1/0.5 vs. 1.0/1.0	6,099	4,654 to 7,545	****	<0,0001
	0.5/0.5 vs. 0.5/1.0	3,473	2,028 to 4,918	****	<0,0001
	0.5/0.5 vs. 1.0/1.0	5,230	3,785 to 6,675	****	<0,0001
	0.5/1.0 vs. 1.0/1.0	1,757	0,3122 to 3,203	**	0,0092
0.750 mg/mL	0.1/0.1 vs 0.1/0.5	0,8222	-0,6229 to 2,267	ns	0,5094
	0.1/0.1 vs 0.5/0.5	-1,845	-3,291 to -0,4003	**	0,0055
	0.1/0.1 vs 0.5/1.0	5,908	4,463 to 7,353	****	<0,0001
	0.1/0.1 vs 1.0/1.0	7,096	5,650 to 8,541	****	<0,0001
	0.1/0.5 vs 0.5/0.5	-2,668	-4,113 to -1,223	****	<0,0001
	0.1/0.5 vs. 0.5/1.0	5,085	3,640 to 6,531	****	<0,0001
	0.1/0.5 vs. 1.0/1.0	6,273	4,828 to 7,719	****	<0,0001

	0.5/0.5 vs. 0.5/1.0	7,753	6,308 to 9,198	****	<0,0001
	0.5/0.5 vs. 1.0/1.0	8,941	7,496 to 10,39	****	<0,0001
	0.5/1.0 vs. 1.0/1.0	1,188	-0,2573 to 2,633	ns	0,1575
1 mg/mL	0.1/0.1 vs 0.1/0.5	-2,800	-4,245 to -1,355	****	<0,0001
	0.1/0.1 vs 0.5/0.5	-0,3685	-1,814 to 1,077	ns	0,9533
	0.1/0.1 vs 0.5/1.0	8,546	7,101 to 9,991	****	<0,0001
	0.1/0.1 vs 1.0/1.0	10,24	8,799 to 11,69	****	<0,0001
	0.1/0.5 vs 0.5/0.5	2,432	0,9864 to 3,877	***	0,0001
	0.1/0.5 vs. 0.5/1.0	11,35	9,901 to 12,79	****	<0,0001
	0.1/0.5 vs. 1.0/1.0	13,04	11,60 to 14,49	****	<0,0001
	0.5/0.5 vs. 0.5/1.0	8,914	7,469 to 10,36	****	<0,0001
	0.5/0.5 vs. 1.0/1.0	10,61	9,167 to 12,06	****	<0,0001
	0.5/1.0 vs. 1.0/1.0	1,698	0,2531 to 3,143	*	0,0130
1.5 mg/mL	0.1/0.1 vs 0.1/0.5	-0,7870	-2,232 to 0,6582	ns	0,5528
	0.1/0.1 vs 0.5/0.5	4,380	2,935 to 5,825	****	<0,0001
	0.1/0.1 vs 0.5/1.0	10,72	9,278 to 12,17	****	<0,0001
	0.1/0.1 vs 1.0/1.0	11,60	10,16 to 13,05	****	<0,0001
	0.1/0.5 vs 0.5/0.5	5,167	3,722 to 6,612	****	<0,0001
	0.1/0.5 vs. 0.5/1.0	11,51	10,07 to 12,96	****	<0,0001
	0.1/0.5 vs. 1.0/1.0	12,39	10,95 to 13,84	****	<0,0001
	0.5/0.5 vs. 0.5/1.0	6,343	4,898 to 7,788	****	<0,0001
	0.5/0.5 vs. 1.0/1.0	7,224	5,779 to 8,669	****	<0,0001
	0.5/1.0 vs. 1.0/1.0	0,8810	-0,5642 to 2,326	ns	0,4390
2 mg/mL	0.1/0.1 vs 0.1/0.5	-2,743	-4,188 to -1,297	****	<0,0001
	0.1/0.1 vs 0.5/0.5	3,134	1,689 to 4,580	****	<0,0001
	0.1/0.1 vs 0.5/1.0	10,41	8,967 to 11,86	****	<0,0001
	0.1/0.1 vs 1.0/1.0	14,45	13,01 to 15,90	****	<0,0001
	0.1/0.5 vs 0.5/0.5	5,877	4,432 to 7,322	****	<0,0001
	0.1/0.5 vs. 0.5/1.0	13,15	11,71 to 14,60	****	<0,0001
	0.1/0.5 vs. 1.0/1.0	17,20	15,75 to 18,64	****	<0,0001
	0.5/0.5 vs. 0.5/1.0	7,278	5,833 to 8,723	****	<0,0001
	0.5/0.5 vs. 1.0/1.0	11,32	9,874 to 12,76	****	<0,0001
	0.5/1.0 vs. 1.0/1.0	4,041	2,596 to 5,486	****	<0,0001

Reducing power

1. Anova

Source	SS	d.o f.	MS	F	p
**Interaction**	2,469	28	0,08817	423,2	P<0,0001
**Row Factor**	2,085	7	0,2979	1430	P<0,0001
**Column Factor**	0,05991	4	0,01498	71,89	P<0,0001
**Residual**	0,01667	80	0,0002083		

2. Tukey's multiple comparisons test

	LS variant	Mean Diff,	95,00% CI of diff,		P Value
0.062	0.1/0.1 vs 0.1/0.5	0,01445	-0,01844 to 0,04734	ns	0,7362
	0.1/0.1 vs 0.5/0.5	0,004550	-0,02834 to 0,03744	ns	0,9952
	0.1/0.1 vs 0.5/1.0	0,01805	-0,01484 to 0,05094	ns	0,5453

	0.1/0.1 vs 1.0/1.0	-0,9802	-1,013 to -0,9473	****	<0,0001
	0.1/0.5 vs 0.5/0.5	-0,009900	-0,04279 to 0,02299	ns	0,9173
	0.1/0.5 vs. 0.5/1.0	0,003600	-0,02929 to 0,03649	ns	0,9981
	0.1/0.5 vs. 1.0/1.0	-0,9947	-1,028 to -0,9618	****	<0,0001
	0.5/0.5 vs. 0.5/1.0	0,01350	-0,01939 to 0,04639	ns	0,7818
	0.5/0.5 vs. 1.0/1.0	-0,9848	-1,018 to -0,9519	****	<0,0001
	0.5/1.0 vs. 1.0/1.0	-0,9983	-1,031 to -0,9654	****	<0,0001
0.125 mg/mL	0.1/0.1 vs 0.1/0.5	0,02005	-0,01284 to 0,05294	ns	0,4390
	0.1/0.1 vs 0.5/0.5	0,0006000	-0,03229 to 0,03349	ns	>0,9999
	0.1/0.1 vs 0.5/1.0	0,008750	-0,02414 to 0,04164	ns	0,9458
	0.1/0.1 vs 1.0/1.0	0,02995	-0,002941 to 0,06284	ns	0,0917
	0.1/0.5 vs 0.5/0.5	-0,01945	-0,05234 to 0,01344	ns	0,4702
	0.1/0.5 vs. 0.5/1.0	-0,01130	-0,04419 to 0,02159	ns	0,8726
	0.1/0.5 vs. 1.0/1.0	0,009900	-0,02299 to 0,04279	ns	0,9173
	0.5/0.5 vs. 0.5/1.0	0,008150	-0,02474 to 0,04104	ns	0,9578
	0.5/0.5 vs. 1.0/1.0	0,02935	-0,003541 to 0,06224	ns	0,1030
	0.5/1.0 vs. 1.0/1.0	0,02120	-0,01169 to 0,05409	ns	0,3814
0.250 mg/mL	0.1/0.1 vs 0.1/0.5	-0,01015	-0,04304 to 0,02274	ns	0,9101
	0.1/0.1 vs 0.5/0.5	-0,008700	-0,04159 to 0,02419	ns	0,9468
	0.1/0.1 vs 0.5/1.0	0,02895	-0,003941 to 0,06184	ns	0,1112
	0.1/0.1 vs 1.0/1.0	0,04765	0,01476 to 0,08054	**	0,0011
	0.1/0.5 vs 0.5/0.5	0,001450	-0,03144 to 0,03434	ns	>0,9999
	0.1/0.5 vs. 0.5/1.0	0,03910	0,006209 to 0,07199	*	0,0116
	0.1/0.5 vs. 1.0/1.0	0,05780	0,02491 to 0,09069	****	<0,0001
	0.5/0.5 vs. 0.5/1.0	0,03765	0,004759 to 0,07054	*	0,0167
	0.5/0.5 vs. 1.0/1.0	0,05635	0,02346 to 0,08924	****	<0,0001
	0.5/1.0 vs. 1.0/1.0	0,01870	-0,01419 to 0,05159	ns	0,5102
0.500 mg/mL	0.1/0.1 vs 0.1/0.5	0,01430	-0,01859 to 0,04719	ns	0,7436
	0.1/0.1 vs 0.5/0.5	-0,01145	-0,04434 to 0,02144	ns	0,8671
	0.1/0.1 vs 0.5/1.0	0,03825	0,005359 to 0,07114	*	0,0144
	0.1/0.1 vs 1.0/1.0	0,07535	0,04246 to 0,1082	****	<0,0001
	0.1/0.5 vs 0.5/0.5	-0,02575	-0,05864 to 0,007141	ns	0,1960
	0.1/0.5 vs. 0.5/1.0	0,02395	-0,008941 to 0,05684	ns	0,2603
	0.1/0.5 vs. 1.0/1.0	0,06105	0,02816 to 0,09394	****	<0,0001
	0.5/0.5 vs. 0.5/1.0	0,04970	0,01681 to 0,08259	***	0,0006
	0.5/0.5 vs. 1.0/1.0	0,08680	0,05391 to 0,1197	****	<0,0001
	0.5/1.0 vs. 1.0/1.0	0,03710	0,004209 to 0,06999	*	0,0191
0.750 mg/mL	0.1/0.1 vs 0.1/0.5	-0,01085	-0,04374 to 0,02204	ns	0,8881
	0.1/0.1 vs 0.5/0.5	0,003300	-0,02959 to 0,03619	ns	0,9986
	0.1/0.1 vs 0.5/1.0	0,01040	-0,02249 to 0,04329	ns	0,9026
	0.1/0.1 vs 1.0/1.0	0,06815	0,03526 to 0,1010	****	<0,0001
	0.1/0.5 vs 0.5/0.5	0,01415	-0,01874 to 0,04704	ns	0,7510
	0.1/0.5 vs. 0.5/1.0	0,02125	-0,01164 to 0,05414	ns	0,3790
	0.1/0.5 vs. 1.0/1.0	0,07900	0,04611 to 0,1119	****	<0,0001
	0.5/0.5 vs. 0.5/1.0	0,007100	-0,02579 to 0,03999	ns	0,9743
	0.5/0.5 vs. 1.0/1.0	0,06485	0,03196 to 0,09774	****	<0,0001
	0.5/1.0 vs. 1.0/1.0	0,05775	0,02486 to 0,09064	****	<0,0001
1	0.1/0.1 vs 0.1/0.5	-0,008250	-0,04114 to 0,02464	****	<0,0001
	0.1/0.1 vs 0.5/0.5	0,03265	-0,0002411 to 0,06554	ns	0,9533

	0.1/0.1 vs 0.5/1.0	0,04205	0,009159 to 0,07494	****	<0,0001
	0.1/0.1 vs 1.0/1.0	0,1182	0,08526 to 0,1510	****	<0,0001
	0.1/0.5 vs 0.5/0.5	0,04090	0,008009 to 0,07379	***	0,0001
	0.1/0.5 vs. 0.5/1.0	0,05030	0,01741 to 0,08319	****	<0,0001
	0.1/0.5 vs. 1.0/1.0	0,1264	0,09351 to 0,1593	****	<0,0001
	0.5/0.5 vs. 0.5/1.0	0,009400	-0,02349 to 0,04229	****	<0,0001
	0.5/0.5 vs. 1.0/1.0	0,08550	0,05261 to 0,1184	****	<0,0001
	0.5/1.0 vs. 1.0/1.0	0,07610	0,04321 to 0,1090	*	0,0130
1.5 mg/mL	0.1/0.1 vs 0.1/0.5	0,05680	0,02391 to 0,08969	****	<0,0001
	0.1/0.1 vs 0.5/0.5	0,06120	0,02831 to 0,09409	****	<0,0001
	0.1/0.1 vs 0.5/1.0	0,04480	0,01191 to 0,07769	**	0,0025
	0.1/0.1 vs 1.0/1.0	0,1747	0,1418 to 0,2075	****	<0,0001
	0.1/0.5 vs 0.5/0.5	0,004400	-0,02849 to 0,03729	ns	0,9958
	0.1/0.5 vs. 0.5/1.0	-0,01200	-0,04489 to 0,02089	ns	0,8462
	0.1/0.5 vs. 1.0/1.0	0,1179	0,08496 to 0,1507	****	<0,0001
	0.5/0.5 vs. 0.5/1.0	-0,01640	-0,04929 to 0,01649	ns	0,6348
	0.5/0.5 vs. 1.0/1.0	0,1135	0,08056 to 0,1463	****	<0,0001
	0.5/1.0 vs. 1.0/1.0	0,1299	0,09696 to 0,1627	****	<0,0001
2 mg/mL	0.1/0.1 vs 0.1/0.5	0,03225	-0,0006411 to 0,06514	ns	0,0574
	0.1/0.1 vs 0.5/0.5	0,07135	0,03846 to 0,1042	****	<0,0001
	0.1/0.1 vs 0.5/1.0	0,04735	0,01446 to 0,08024	**	0,0012
	0.1/0.1 vs 1.0/1.0	0,1882	0,1553 to 0,2210	****	<0,0001
	0.1/0.5 vs 0.5/0.5	0,03910	0,006209 to 0,07199	*	0,0116
	0.1/0.5 vs. 0.5/1.0	0,01510	-0,01779 to 0,04799	ns	0,7034
	0.1/0.5 vs. 1.0/1.0	0,1559	0,1230 to 0,1888	****	<0,0001
	0.5/0.5 vs. 0.5/1.0	-0,02400	-0,05689 to 0,008891	ns	0,2584
	0.5/0.5 vs. 1.0/1.0	0,1168	0,08391 to 0,1497	****	<0,0001
	0.5/1.0 vs. 1.0/1.0	0,1408	0,1079 to 0,1737	****	<0,0001

Chelating activity (%)

1. Anova

Source	SS	d.o f.	MS	F	p
**Interaction**	7462	28	266,5	201,4	P<0,0001
**Row Factor**	74583	7	10655	8052	P<0,0001
**Column Factor**	13204	4	3301	2495	P<0,0001
**Residual**	105,9	80	1,323		

2. Tukey's multiple comparisons test

	LS variant	Mean Diff,	95,00% CI of diff,		P Value
0.0625 mg/mL	0.1/0.1 vs 0.1/0.5	0,000	-2,621 to 2,621	ns	>0,9999
	0.1/0.1 vs 0.5/0.5	0,000	-2,621 to 2,621	ns	>0,9999
	0.1/0.1 vs 0.5/1.0	0,000	-2,621 to 2,621	ns	>0,9999
	0.1/0.1 vs 1.0/1.0	0,000	-2,621 to 2,621	ns	>0,9999
	0.1/0.5 vs 0.5/0.5	0,000	-2,621 to 2,621	ns	>0,9999
	0.1/0.5 vs. 0.5/1.0	0,000	-2,621 to 2,621	ns	>0,9999
	0.1/0.5 vs. 1.0/1.0	0,000	-2,621 to 2,621	ns	>0,9999
	0.5/0.5 vs. 0.5/1.0	0,000	-2,621 to 2,621	ns	>0,9999
	0.5/0.5 vs. 1.0/1.0	0,000	-2,621 to 2,621	ns	>0,9999

	0.5/1.0 vs. 1.0/1.0	0,000	-2,621 to 2,621	ns	>0,9999
0.125 mg/mL	0.1/0.1 vs 0.1/0.5	0,000	-2,621 to 2,621	ns	>0,9999
	0.1/0.1 vs 0.5/0.5	-1,487	-4,108 to 1,135	ns	0,5126
	0.1/0.1 vs 0.5/1.0	-6,513	-9,134 to -3,892	****	<0,0001
	0.1/0.1 vs 1.0/1.0	0,000	-2,621 to 2,621	ns	>0,9999
	0.1/0.5 vs 0.5/0.5	-1,487	-4,108 to 1,135	ns	0,5126
	0.1/0.5 vs. 0.5/1.0	-6,513	-9,134 to -3,892	****	<0,0001
	0.1/0.5 vs. 1.0/1.0	0,000	-2,621 to 2,621	ns	>0,9999
	0.5/0.5 vs. 0.5/1.0	-5,026	-7,648 to -2,405	****	<0,0001
	0.5/0.5 vs. 1.0/1.0	1,487	-1,135 to 4,108	ns	0,5126
	0.5/1.0 vs. 1.0/1.0	6,513	3,892 to 9,134	****	<0,0001
0.250 mg/mL	0.1/0.1 vs 0.1/0.5	8,397	5,776 to 11,02	****	<0,0001
	0.1/0.1 vs 0.5/0.5	2,733	0,1121 to 5,355	*	0,0367
	0.1/0.1 vs 0.5/1.0	-7,518	-10,14 to -4,897	****	<0,0001
	0.1/0.1 vs 1.0/1.0	4,935	2,314 to 7,557	****	<0,0001
	0.1/0.5 vs 0.5/0.5	-5,664	-8,285 to -3,043	****	<0,0001
	0.1/0.5 vs. 0.5/1.0	-15,92	-18,54 to -13,29	****	<0,0001
	0.1/0.5 vs. 1.0/1.0	-3,462	-6,083 to -0,8406	**	0,0037
	0.5/0.5 vs. 0.5/1.0	-10,25	-12,87 to -7,631	****	<0,0001
	0.5/0.5 vs. 1.0/1.0	2,202	-0,4193 to 4,823	ns	0,1418
	0.5/1.0 vs. 1.0/1.0	12,45	9,832 to 15,08	****	<0,0001
0.500 mg/mL	0.1/0.1 vs 0.1/0.5	26,30	23,68 to 28,92	****	<0,0001
	0.1/0.1 vs 0.5/0.5	15,23	12,61 to 17,85	****	<0,0001
	0.1/0.1 vs 0.5/1.0	-2,878	-5,500 to -0,2570	*	0,0241
	0.1/0.1 vs 1.0/1.0	2,817	0,1961 to 5,439	*	0,0288
	0.1/0.5 vs 0.5/0.5	-11,07	-13,69 to -8,447	****	<0,0001
	0.1/0.5 vs. 0.5/1.0	-29,18	-31,80 to -26,55	****	<0,0001
	0.1/0.5 vs. 1.0/1.0	-23,48	-26,10 to -20,86	****	<0,0001
	0.5/0.5 vs. 0.5/1.0	-18,11	-20,73 to -15,49	****	<0,0001
	0.5/0.5 vs. 1.0/1.0	-12,41	-15,03 to -9,790	****	<0,0001
	0.5/1.0 vs. 1.0/1.0	5,696	3,074 to 8,317	****	<0,0001
0.750 mg/mL	0.1/0.1 vs 0.1/0.5	32,10	29,47 to 34,72	****	<0,0001
	0.1/0.1 vs 0.5/0.5	13,85	11,23 to 16,47	****	<0,0001
	0.1/0.1 vs 0.5/1.0	-2,575	-5,197 to 0,04610	ns	0,0566
	0.1/0.1 vs 1.0/1.0	-6,832	-9,453 to -4,211	****	<0,0001
	0.1/0.5 vs 0.5/0.5	-18,25	-20,87 to -15,62	****	<0,0001
	0.1/0.5 vs. 0.5/1.0	-34,67	-37,29 to -32,05	****	<0,0001
	0.1/0.5 vs. 1.0/1.0	-38,93	-41,55 to -36,31	****	<0,0001
	0.5/0.5 vs. 0.5/1.0	-16,42	-19,05 to -13,80	****	<0,0001
	0.5/0.5 vs. 1.0/1.0	-20,68	-23,30 to -18,06	****	<0,0001
	0.5/1.0 vs. 1.0/1.0	-4,257	-6,878 to -1,636	***	0,0002
1 mg/mL	0.1/0.1 vs 0.1/0.5	43,03	40,41 to 45,65	****	<0,0001
	0.1/0.1 vs 0.5/0.5	27,97	25,35 to 30,59	****	<0,0001
	0.1/0.1 vs 0.5/1.0	0,2503	-2,371 to 2,872	ns	0,9989
	0.1/0.1 vs 1.0/1.0	-4,651	-7,273 to -2,030	****	<0,0001
	0.1/0.5 vs 0.5/0.5	-15,06	-17,68 to -12,43	****	<0,0001
	0.1/0.5 vs. 0.5/1.0	-42,78	-45,40 to -40,16	****	<0,0001
	0.1/0.5 vs. 1.0/1.0	-47,68	-50,30 to -45,06	****	<0,0001
	0.5/0.5 vs. 0.5/1.0	-27,72	-30,34 to -25,10	****	<0,0001

	0.5/0.5 vs. 1.0/1.0	-32,62	-35,24 to -30,00	****	<0,0001
	0.5/1.0 vs. 1.0/1.0	-4,902	-7,523 to -2,280	****	<0,0001
1.5 mg/mL	0.1/0.1 vs 0.1/0.5	47,41	44,79 to 50,03	****	<0,0001
	0.1/0.1 vs 0.5/0.5	30,13	27,51 to 32,75	****	<0,0001
	0.1/0.1 vs 0.5/1.0	2,748	0,1268 to 5,369	*	0,0352
	0.1/0.1 vs 1.0/1.0	-4,592	-7,213 to -1,971	****	<0,0001
	0.1/0.5 vs 0.5/0.5	-17,28	-19,90 to -14,66	****	<0,0001
	0.1/0.5 vs. 0.5/1.0	-44,66	-47,28 to -42,04	****	<0,0001
	0.1/0.5 vs. 1.0/1.0	-52,00	-54,62 to -49,38	****	<0,0001
	0.5/0.5 vs. 0.5/1.0	-27,38	-30,00 to -24,76	****	<0,0001
	0.5/0.5 vs. 1.0/1.0	-34,72	-37,34 to -32,10	****	<0,0001
	0.5/1.0 vs. 1.0/1.0	-7,340	-9,962 to -4,719	****	<0,0001
2 mg/mL	0.1/0.1 vs 0.1/0.5	38,52	35,90 to 41,14	****	<0,0001
	0.1/0.1 vs 0.5/0.5	23,17	20,55 to 25,79	****	<0,0001
	0.1/0.1 vs 0.5/1.0	3,186	0,5642 to 5,807	**	0,0093
	0.1/0.1 vs 1.0/1.0	-3,880	-6,501 to -1,258	***	0,0008
	0.1/0.5 vs 0.5/0.5	-15,35	-17,97 to -12,73	****	<0,0001
	0.1/0.5 vs. 0.5/1.0	-35,33	-37,96 to -32,71	****	<0,0001
	0.1/0.5 vs. 1.0/1.0	-42,40	-45,02 to -39,78	****	<0,0001
	0.5/0.5 vs. 0.5/1.0	-19,99	-22,61 to -17,37	****	<0,0001
	0.5/0.5 vs. 1.0/1.0	-27,05	-29,67 to -24,43	****	<0,0001
	0.5/1.0 vs. 1.0/1.0	-7,065	-9,686 to -4,444	****	<0,0001
